# Supplementary material for: Activation of the PI3K/AKT/mTOR Pathway in Cajal–Retzius Cells Leads to Their Survival and Increases Susceptibility to Kainate-Induced Seizures
Source: Int J Mol Sci. 2023 Mar 11;24(6):5376. doi: 10.3390/ijms24065376 (PMC10048971; doi:10.3390/ijms24065376)
Supplement: Supplementary file 1 [file ijms-24-05376-s001.zip › AllTablesSup.pdf]

ROSTRO-CAUDAL AXE

Quantification of total number of CR in three neocortex levels (Table 1.1)

Mann-Whitney U test	*Two-tailed p* value	U	N	E17.5) Mean (10) ± SD (10	(P1) Mean (10) ± SD (10)	Significant (alpha=0.05)?	P value summary
Level 1- E17.5 vs P1	0,0286	0	4	20.8 ± 2.67	12.0 ± 2.04	Yes	*
Level 2- E17.5 vs P1	0,6857	6	4	15.7 ± 1.98	15.7 ± 2.07	No	ns
Level 3- E17.5 vs P1	0,4571	5	4	16.2 ± 2.88	14.8 ± 2.70	No	ns

Quantification of CR density (CRs/mm3) in three neocortex levels (Table 1.2)

Mann-Whitney U test	*Two-tailed p* value	U	N	(E17.5) Mean ± SD (10^3)	(P1) Mean ± SD (10^3)	Significant (alpha=0.05)?	P value summary
Level 1- E17.5 vs P1	0,0286	0	4	25.5 ± 1.65	14.1 ± 2.43	Yes	*
Level 2- E17.5 vs P1	0,0286	0	4	23.4 ± 2.47	14.7 ± 2.12	Yes	*
Level 3- E17.5 vs P1	0,0286	0	4	30.0 ± 2.58	15.4 ± 2.64	Yes	*

Quantification of the proportion of CRs for the PI3K/AKT/mTOR activity over total tdTOMATO cells in three neocortex levels (Table 1.3)

Mann-Whitney U test	*Two-tailed p* value	U	N	(E17.5) Mean ± SD	(P1) Mean ± SD	Significant (alpha=0.05)?	P value summary
CRs positive only for pS6 over total tdTOMATO cells							
Level 1- E17.5 vs P1	0.4857	5	4	18.7 ± 2.43	15.3 ± 4.86	No	ns
Level 2- E17.5 vs P1	0.4857	5	4	18.8 ± 7.80	14.5 ± 3.69	No	ns
Level 3- E17.5 vs P1	0.0571	1	4	18.8 ± 6.90	10.8 ± 2.04	No	ns
CRs positive only for pAKT over total tdTOMATO cells							
Level 1- E17.5 vs P1	0.3429	4	4	23.2 ± 6.20	17.2 ± 8.04	No	ns
Level 2- E17.5 vs P1	0.6857	6	4	26.2 ± 8.95	23.7 ± 6.00	No	ns
Level 3- E17.5 vs P1	0.8857	7	4	22.3 ± 3.23	25.2 ± 8.93	No	ns
CRs positive for both pS6 & pAKT over total tdTOMATO cells							
Level 1- E17.5 vs P1	0.0286	0	4	21.4 ± 7.45	8.6 ± 3.55	Yes	*
Level 2- E17.5 vs P1	0.0286	0	4	24.4 ± 4.98	9.0 ± 3.37	Yes	*
Level 3- E17.5 vs P1	0.0286	0	4	30.0 ± 7.46	9.4 ± 2.94	Yes	*
CRs negative for any activity over total tdTOMATO cells							
Level 1- E17.5 vs P1	0.0286	0	4	36.5 ± 7.4	58.8 ± 5.7	Yes	*
Level 2- E17.5 vs P1	0.0286	0	4	30.3 ± 4.50	52.6 ± 9.06	Yes	*
Level 3- E17.5 vs P1	0.0286	0	4	28.6 ± 2.24	54.4 ± 12.07	Yes	*

MEDIO-LATERAL AXE in three neocortex levels

Analysis of the PI3K/AKT/mTOR Pathway activity distribution along the medio-lateral axis in three neocortex levels at E17.5 and P1 (Table 1.4 and Table 1.5)

	E17.5 (Table 1.4)					P1 (Table 1.5)				
Kolmogorov-Smirnov test	*p* value	Distance	N	Significant (alpha=0.05)?	P value summary	*p* value	Distance	N	Significant (alpha=0.05)?	P value summary
pS6 vs tdT										
Level 1	<0.0001	0.2943	4	Yes	****	0.7833	0,0828	4	No	ns
Level 2	0.0006	0.2043	4	Yes	***	0.1294	0.1312	4	No	ns
Level 3	0.0047	0.1707	4	Yes	**	<0.0001	0.294	4	Yes	****
pAKT vs tdT										
Level 1	0.0094	0.1308	4	Yes	**	0.0577	0.1583	4	No	ns
Level 2	0.0001	0.1911	4	Yes	***	<0.0001	0.2105	4	Yes	****
Level 3	<0.0001	0.2086	4	Yes	****	0.0018	0.1696	4	Yes	**
pS6 & pAKT vs tdT										
Level 1	0.0069	0.1401	4	Yes	**	0.003	0.2967	4	Yes	**
Level 2	0.226	0.09316	4	No	ns	0.0174	0.2148	4	Yes	*
Level 3	0.0401	0.1154	4	Yes	*	0.4064	0.1225	4	No	ns

Quantification of CR density (CRs/mm3) at P1 stage in three neocortex levels

Table 2.1

	Control	PIK3CA	TSC1	PTEN
	Mean (10^3) ± SD (10^3)	Mean (10^3)± SD (10^3)	Mean(10^3) ± SD (10^3)	Mean(10^3) ± SD (10^3)
Level 1	14.1 ± 2.43	10.6 ± 1.06	11.0 ± 3.89	8.1 ± 3.35
Level 2	14.7 ± 2.12	10.9 ± 3.00	9.9 ± 2.79	8.7 ± 3.59
Level 3	15.4 ± 2.64	11.9 ± 4.98	10.2 ± 4.41	10.1 ± 6.37

Table 2.2

Two way ANOVA table	SS	DF	MS	F (DFn. DFd)	P value	Summary	Significant?
**control vs PIK3CA**							
Interaction	135,9E+3	2,0E+0	67,9E+3	F (2, 12) = 0.01026	989,8E-3	ns	No
levels	7,7E+6	2,0E+0	3,8E+6	F (2, 12) = 0.5810	574,3E-3	ns	No
Genotype	76,7E+6	1,0E+0	76,7E+6	F (1, 6) = 5.945	50,6E-3	ns	No
Subject	77,4E+6	6,0E+0	12,9E+6	F (6, 12) = 1.949	153,2E-3	ns	No
Residual	79,5E+6	12,0E+0	6,6E+6				
**control vs TSC1**							
Interaction	5,2E+6	2,0E+0	2,6E+6	F (2, 12) = 0.5317	600,8E-3	ns	No
levels	996,9E+3	2,0E+0	498,5E+3	F (2, 12) = 0.1026	903,3E-3	ns	No
Genotype	114,1E+6	1,0E+0	114,1E+6	F (1, 6) = 5.653	55,0E-3	ns	No
Subject	121,1E+6	6,0E+0	20,2E+6	F (6, 12) = 4.155	17,2E-3	*	Yes
Residual	58,3E+6	12,0E+0	4,9E+6				
**control vs PTEN**							
Interaction	555,2E+3	2,0E+0	277,6E+3	F (2, 12) = 0.04847	952,9E-3	ns	No
levels	12,0E+6	2,0E+0	6,0E+6	F (2, 12) = 1.047	380,9E-3	ns	No
Genotype	198,6E+6	1,0E+0	198,6E+6	F (1, 6) = 6.711	41,2E-3	*	Yes
Subject	177,5E+6	6,0E+0	29,6E+6	F (6, 12) = 5.167	7,7E-3	**	Yes
Residual	68,7E+6	12,0E+0	5,7E+6				

Sidak's multiple comparisons test	Mean 1 (Control)	Mean 2 (Mutant)	Mean Diff.	SE of diff.	95.00% CI of diff.	N1	N2	t	DF	justed P Val	Summary	Significant?
Level 1												
Control vs. PIK3CA	14055	10606	3449	2577	-4805 to 11704	4	4	1.338	36	0.9771	ns	No
Control vs. TSC1	14055	10972	3083	2577	-5172 to 11337	4	4	1.196	36	0.9928	ns	No
Control vs. PTEN	14055	8072	5983	2577	-2272 to 14238	4	4	2.321	36	0.3781	ns	No
Level 2												
Control vs. PIK3CA	14685	10898	3787	2577	-4467 to 12042	4	4	1.469	36	0.9468	ns	No
Control vs. TSC1	14685	9944	4742	2577	-3513 to 12997	4	4	1.84	36	0.7496	ns	No
Control vs. PTEN	14685	8733	5953	2577	-2302 to 14207	4	4	2.31	36	0.3862	ns	No
Level 3												
Control vs. PIK3CA	15439	11948	3491	2577	-4764 to 11746	4	4	1.355	36	0.9743	ns	No
Control vs. TSC1	15439	10182	5257	2577	-2997 to 13512	4	4	2.04	36	0.5933	ns	No
Control vs. PTEN	15439	10116	5323	2577	-2932 to 13578	4	4	2.065	36	0.5728	ns	No

Table 2.3

Quantification of the proportion of CRs for the PI3K/AKT/mTOR activity over total tdTOMATO cells in three neocortex levels at P1 stage

Mann-Whitney U test	*Two-tailed p* value	U	N (Control and Mutants)	Mean ± SD **Control**	Mean ± SD **Mutant**	Significant (alpha=0.05)?	P value summary
CRs positive only for pS6 over total tdTOMATO cells P1 stage							
Level 1							
Control vs. PIK3CA	0.1143	2	4	15.5 ± 4.57	8.2±4.21	No	ns
Control vs. TSC1	0.6857	6	4	15.5 ± 4.57	16.7± 9.78	No	ns
Control vs. PTEN	0.3429	4	4	15.5 ± 4.57	10.3±3.84	No	ns
Level 2							
Control vs. PIK3CA	0.1143	2	4	14.0 ± 3.58	9.0±3.01	No	ns
Control vs. TSC1	0.1143	2	4	14.0 ± 3.58	21.8±5.33	No	ns
Control vs. PTEN	0.4571	5	4	14.0 ± 3.58	10.2±5.75	No	ns
Level 3							
Control vs. PIK3CA	0.6857	6	4	10.8 ± 2.04	10.2±4.84	No	ns
Control vs. TSC1	0.0286	0	4	10.8 ± 2.04	24.0± 5.52	Yes	*
Control vs. PTEN	0.2	3	4	10.8 ± 2.04	6.5±3.63	No	ns
CRs positive only for pAKT over total tdTOMATO cells P1 stage							
Level 1							
Control vs. PIK3CA	0.8857	7	4	17.4 ± 8.4	17.0±3.94	No	ns
Control vs. TSC1	0.4857	5	4	17.4 ± 8.4	22.9± 9.95	No	ns
Control vs. PTEN	0.0286	0	4	17.4 ± 8.4	6.7±3.05	Yes	*
Level 2							
Control vs. PIK3CA	0.2	3	4	23.1 ± 5.26	14.3±8.62	No	ns
Control vs. TSC1	0.0286	0	4	23.1 ± 5.26	9.5±5.26	Yes	*
Control vs. PTEN	0.0571	1	4	23.1 ± 5.26	12.7±6.67	No	ns
Level 3							
Control vs. PIK3CA	0.4857	5	4	25.5 ± 9.08	17.9±18.7	No	ns
Control vs. TSC1	0.4857	5	4	25.5 ± 9.08	16.6± 11.4	No	ns
Control vs. PTEN	0.3429	4	4	25.5 ± 9.08	15.6±13.90	No	ns
CRs positive for both pS6 & pAKT over total tdTOMATO cells P1 stage							
Level 1							
Control vs. PIK3CA	0.0286	0	4	8.6 ± 3.55	38.1±15.3	Yes	*
Control vs. TSC1	0.0571	1	4	8.6 ± 3.55	16.3± 4.53	No	ns
Control vs. PTEN	0.8857	7	4	8.6 ± 3.55	11.7±8.49	No	ns
Level 2							
Control vs. PIK3CA	0.0286	0	4	9.04 ± 3.37	24.8±16.09	Yes	*
Control vs. TSC1	0.8857	7	4	9.04 ± 3.37	12.2±10.27	No	ns
Control vs. PTEN	0.2	3	4	9.04 ± 3.37	22.2±14.67	No	ns
Level 3							
Control vs. PIK3CA	0.0286	0	4	9.4 ± 2.94	38.5±13.58	Yes	*
Control vs. TSC1	0.0286	0	4	9.4 ± 2.94	20.5± 10.15	Yes	*
Control vs. PTEN	0.0286	0	4	9.4 ± 2.94	37.0±18.29	Yes	*
CRs negative for any activity over total tdTOMATO cells P1 stage							
Level 1							
Control vs. PIK3CA	0.0286	0	4	58.3 ± 6.07	36.4±11.73	Yes	*
Control vs. TSC1	0.0286	0	4	58.3 ± 6.07	43.9± 4.21	Yes	*
Control vs. PTEN	0.2	3	4	58.3 ± 6.07	71.0±13.01	No	ns
Level 2							
Control vs. PIK3CA	0.6857	6	4	53.7 ± 8.11	51.7±17.71	No	ns
Control vs. TSC1	0.6857	6	4	53.7 ± 8.11	56.4±9.30	No	ns
Control vs. PTEN	0.8857	7	4	53.7 ± 8.11	54.7±17.9	No	ns
Level 3							
Control vs. PIK3CA	0.1143	2	4	54.1 ±12.13	33.22±20.25	No	ns
Control vs. TSC1	0.3429	4	4	54.1 ±12.13	38.8± 15.32	No	ns
Control vs. PTEN	0.4857	5	4	54.1 ±12.13	40.7±20.3	No	ns

Table 2.4

Quantification of the neocortex thickness (µm) of control and mutants in the futur S1 brain area at P1 stage (level 2)			
Unpaired t test	PIK3CA Vs Control	TSC1 Vs Control	PTEN Vs Control
P value	0,0343	0,218	0,0412
P value summary	*	ns	*
Significantly different (P < 0.05)?	Yes	No	Yes
One- or two-tailed P value?	Two-tailed	Two-tailed	Two-tailed
t, df	t=2.727, df=6	t=1.409, df=5	t=2.590, df=6
How big is the difference?			
Mean of control	548,8	548,8	548,8
Mean of mutant	651,8	575	627,5
Difference between means ± SEM	103.0 ± 37.76	26.25 ± 18.64	78.75 ± 30.40
95% confidence interval	10.59 to 195.4	-21.65 to 74.15	4.358 to 153.1
R squared (eta squared)	0,5535	0,2841	0,5279
F test to compare variances			
F, DFn, Dfd	9.122, 3, 3	1.141, 2, 3	5.560, 3, 3
P value	0,1023	0,8561	0,1926
P value summary	ns	ns	ns
Significantly different (P < 0.05)?	No	No	No
Data analyzed			
Sample size, Control	4	4	4
Sample size, mutant	4	3	4

ROSTRO-CAUDAL AXE

Quantification of CR density (CRs/mm3) at P24 stage in three neocortex levels (Table 3.1)

	Control	PIK3CA	TSC1	PTEN
	Mean ± SD	Mean ± SD	Mean ± SD	Mean ± SD
Level 1	362.7 ± 111.4	957.0 ± 220.2	633.8 ± 84.5	1322.0 ± 508.9
Level 2	273.0 ± 97.1	1134.3 ± 82.0	553.4± 207.2	1816.4 ± 566.7
Level 3	556.7 ± 138.5	1472.2± 271.8	1054.3± 233.8	2022.5 ± 733.7

control vs PIK3CA

Two Way ANOVA table	SS	DF	MS	F (DFn, DFd)	P value	Summary	Significant?
Interaction	131369	2	65684	F (2, 14) = 4.074	0,0403	*	Yes
level	664455	2	332228	F (2, 14) = 20.61	<0.0001	****	Yes
Genotype	4164352	1	4164352	F (1, 7) = 89.42	<0.0001	****	Yes
Subjects (matching)	325989	7	46570	F (7, 14) = 2.888	0,0433	*	Yes
Residual	225730	14	16124				

Sidak's multiple comparisons test	Mean 1 (control)	Mean 2 (PIK3CA)	Mean Diff.	SE of diff.	95.00% CI of diff.	N1 (control)	N2 (PIK3CA)	t	DF	Adjusted P Value	Summary	Significant?
Delta Np73$^{Cre/+}$ - Delta Np73 $^{Cre/+}$> R26 $^{p110*/tdT}$												
Level1 (control vs PIK3CA)	362.8	957.1	-594.3	108.7	-876.3 to -312.3	5	4	5.466	21	<0.0001	****	Yes
Level2 (control vs PIK3CA)	273.1	1134	-861.3	108.7	-1143 to -579.3	5	4	7.921	21	<0.0001	****	Yes
Level3 (control vs PIK3CA)	556.8	1472	-915.5	108.7	-1197 to -633.5	5	4	8.42	21	<0.0001	****	Yes

control vs TSC1

Two Way ANOVA table	SS	DF	MS	F (DFn, DFd)	P value	Summary	Significant?
Interaction	73024	2	36512	F (2, 14) = 4.533	0,0304	*	Yes
Level	757233	2	378617	F (2, 14) = 47	<0.0001	****	Yes
Genotype	815284	1	815284	F (1, 7) = 15.6	0,0055	**	Yes
Subjects (matching)	365758	7	52251	F (7, 14) = 6.487	0,0015	**	Yes
Residual	112774	14	8055				

Sidak's multiple comparisons test	Mean 1 (control)	Mean 2 (TSC1)	Mean Diff.	SE of diff.	95.00% CI of diff.	N1 (control)	N2 (TSC1)	t	DF	Adjusted P Value	Summary	Significant?
Delta Np73$^{Cre/+}$ - Delta Np73Cre/+>TSC1lox/del												
Level1 (control vs TSC1)	362.8	633.9	-271.1	101.3	-533.8 to -8.492	5	4	2.677	21	0.0417	*	Yes
Level2 (control vs TSC1)	273.1	553.4	-280.4	101.3	-543 to -17.73	5	4	2.769	21	0.0341	*	Yes
Level3 (control vs TSC1)	556.8	1054	-497.6	101.3	-760.3 to -235	5	4	4.914	21	0.0002	***	Yes

control vs PTEN*

Multiple t-test	Mean1 (control)	Mean2 (PTEN)	Difference	SE of difference	t ratio	df	N1 (control)	N2 (PTEN)	P value	Summary	Significant?
Level1 (control vs PTEN)	362.8	1322	959.3	234.5	4.091	10	5	7	0.0022	##	Yes
Level2 (control vs PTEN)	273.1	1816	1543	259.6	5.946	10	5	7	0.0001	###	Yes
Level3 (control vs PTEN)	556.8	2023	1466	336.7	4.353	10	5	7	0.0014	##	Yes

TSC1 vs PTEN*

Multiple t-test	Mean1 PTEN	Mean2 TSC1	Difference	SE of difference	t ratio	df	N1 (PTEN)	N2 (TSC1)	P value	Summary	Significant?
Level1 (PTEN vs TSC1)	1322	633.9	688.2	262.3	2.624	9	7	4	0,0276	*	Yes
Level2 (PTEN vs TSC1)	1816	553.4	1263	299.6	4.215	9	7	4	0,0022	**	Yes
Level3 (PTEN vs TSC1)	2023	1054	968.2	384.9	2.515	9	7	4	0,033	*	Yes

* Due to inequal variance (larger variation in pten mutants) we used mutiple-t-test to compare Pten data to either control ot Tsc1

MEDIO-LATERAL AXE in three neocortex levels

Quantification of CR density (CRs/mm3) at P24 along medio-lateral and rostro-caudal axes (Table 3.2)

control vs PIK3CA

Two ways ANOVA table	SS	DF	MS	F (DFn, DFd)	P value	Summary	Significant?
Level 1							
Interaction	1221622	3	407207	F (3, 21) = 5.353	0,0068	**	Yes
Lateral axe	4351360	3	1450453	F (3, 21) = 19.07	<0.0001	****	Yes
Genotype	4002641	1	4002641	F (1, 7) = 22.05	0,0022	**	Yes
Subjects (matching)	1270643	7	181520	F (7, 21) = 2.386	0,0581	ns	No
Residual	1597545	21	76074				
Level 2							
Interaction	1175038	3	391679	F (3, 21) = 29.31	<0.0001	****	Yes
Lateral Axe	3190997	3	1063666	F (3, 21) = 79.6	<0.0001	****	Yes
Genotype	6357467	1	6357467	F (1, 7) = 178.3	<0.0001	****	Yes
Subjects (matching)	249613	7	35659	F (7, 21) = 2.669	0,0384	*	Yes
Residual	280613	21	13363				
Level 3							
Interaction	1212739	2	606369	F (2, 14) = 6.746	0,0089	**	No
Lateral Axe	6063707	2	3031854	F (2, 14) = 33.73	<0.0001	****	Yes
Genotype	5026709	1	5026709	F (1, 7) = 30.13	0,0009	***	Yes
Subjects (matching)	1167724	7	166818	F (7, 14) = 1.856	0,1538	ns	No
Residual	1258330	14	89881				

Sidak's multiple comparisons test	Mean 1 (control)	Mean 2 (PIK3CA)	Mean Diff.	SE of diff.	95.00% CI of diff.	N1 (control)	N2 (PIK3CA)	t	DF	Adjusted P Value	Summary	Significant?
Level 1												
ACA (control vs PIK3CA)	980	1349	-369.4	214.7	-940.8 to 202	5	4	1.721	28	0.3332	ns	No
MOs (control vs PIK3CA)	369	1541	-1172	214.7	-1743 to -600.3	5	4	5.457	28	<0.0001	****	Yes
MOp (control vs PIK3CA)	201.2	1080	-878.4	214.7	-1450 to -307	5	4	4.091	28	0.0013	**	Yes
SSp (control vs PIK3CA)	104.3	369	-264.7	214.7	-836.1 to 306.7	5	4	1.233	28	0.6445	ns	No
Level 2												
RSP-MO (control vs PIK3CA)	467.5	1866	-1398	92.31	-1644 to -1152	5	4	15.15	28	<0.0001	****	Yes
SSp (control vs PIK3CA)	247.9	1192	-943.9	92.31	-1190 to -698.2	5	4	10.22	28	<0.0001	****	Yes
SSp-bfd(control vs PIK3CA)	150.1	636.6	-486.4	92.31	-732.1 to -240.8	5	4	5.27	28	<0.0001	****	Yes
SSs-AUD (control vs PIK3CA)	205.5	759.8	-554.4	92.31	-800.1 to -308.7	5	4	6.005	28	<0.0001	****	Yes
Level 3												
RSP(control vs PIK3CA)	945.7	2356	-1410	228	-2002 to -819	5	4	6.186	21	<0.0001	****	Yes
VIS (control vs PIK3CA)	242.9	1069	-826.6	228	-1418 to -235.2	5	4	3.625	21	0.0048	**	Yes
AUD (control vs PIK3CA)	439.3	807.4	-368.1	228	-959.5 to 223.2	5	4	1.614	21	0.3217	ns	No

control vs TSC1

Two ways ANOVA table	SS	DF	MS	F (DFn, DFd)	P value	Summary	Significant?
Level 1							
Interaction	565981	3	188660	F (3, 21) = 5.459	0,0062	***	Yes
Lateral axe	7267043	3	2422348	F (3, 21) = 70.09	<0.0001	****	Yes
Genotype	583725	1	583725	F (1, 7) = 8.285	0,0237	*	Yes
Subjects (matching)	493194	7	70456	F (7, 21) = 2.039	0,0977	ns	No
Residual	725786	21	34561				
Level 2							
Interaction	217565	3	72522	F (3, 21) = 3.332	0,0391	*	Yes
Lateral axe	971351	3	323784	F (3, 21) = 14.88	<0.0001	****	Yes
Genotype	628213	1	628213	F (1, 7) = 6.887	0,0342	*	Yes
Subjects (matching)	638561	7	91223	F (7, 21) = 4.191	0,0049	**	Yes
Residual	457044	21	21764				
Level 3							
Interaction	32517	2	16258	F (2, 14) = 0.2499	0,7823	ns	No
Lateral Axe	2910455	2	1455227	F (2, 14) = 22.37	<0.0001	****	Yes
Genotype	1694583	1	1694583	F (1, 7) = 17.97	0,0038	**	Yes
Subjects (matching)	660285	7	94326	F (7, 14) = 1.45	0,262	ns	No
Residual	910860	14	65061				

Sidak's multiple comparisons test	Mean 1 (control)	Mean 2 (TSC1)	Mean Diff.	SE of diff.	95.00% CI of diff.	N1 (control)	N2 (TSC1)	t	DF	Adjusted P Value	Summary	Significant?
Delta Np73$^{Cre/+}$ - Delta Np73Cre/+>TSC1lox/del												
Level 1												
ACA (control vs TSC1)	980	1656	-675.6	140	-1048 to -303.1	5	4	4.827	28	0.0002	***	Yes
MOs (control vs TSC1)	369	371.4	-2.356	140	-374.9 to 370.1	5	4	0.01683	28	>0.9999	ns	No
MOp(control vs TSC1)	201.2	356.3	-155.1	140	-527.6 to 217.4	5	4	1.108	28	0.7271	ns	No
SSp (control vs TSC1)	104.3	296.2	-192	140	-564.5 to 180.5	5	4	1.371	28	0.5504	ns	No
Level 2												
RSP-MO (control vs TSC1)	467.5	895.7	-428.2	132.7	-781.4 to -75.1	5	4	3.227	28	0.0126	*	Yes
SSp (control vs TSC1)	247.9	262.7	-14.81	132.7	-368 to 338.3	5	4	0.1116	28	>0.9999	ns	No
SSp-bfd(control vs TSC1)	150.1	508.2	-358	132.7	-711.2 to -4.874	5	4	2.698	28	0.0459	*	Yes
SSs-AUD (control vs TSC1)	205.5	467.8	-262.3	132.7	-615.5 to 90.85	5	4	1.977	28	0.2126	ns	No
Level 3												
RSP (control vs TSC1)	945.7	1548	-602.7	183.5	-1079 to -126.8	5	4	3.285	21	0.0106	*	Yes
VIS (control vs TSC1)	242.9	691.9	-449	183.5	-924.9 to 26.88	5	4	2.447	21	0.0682	ns	No
AUD (control vs TSC1)	439.3	900.1	-460.8	183.5	-936.7 to 15.09	5	4	2.511	21	0.0596	ns	No

control vs PTEN*

Multiple t-test	Mean 1 (control)	Mean 2 (PTEN)	Difference	SE of difference	t ratio	df	P value	Summary	Significant?
Level 1									
ACA (control vs PTEN)	980	3005	2025	582.4	3.476	10	0,0059	##	Yes
MOs (control vs PTEN)	369	1084	714.5	258.8	2.761	10	0,0201	#	Yes
MOp (control vs PTEN)	201.2	821.9	620.7	233.8	2.655	10	0,0241	#	Yes
SSp (control vs PTEN)	104.3	666.1	561.8	128.8	4.361	10	0,0014	##	Yes
Level 2									
RSP-MO (control vs PTEN)	205.5	2057	1851	234.1	7.909	10	<0.0001	####	Yes
SSp (control vs PTEN)	150.1	1276	1126	219.1	5.137	10	0,0004	###	Yes
SSp-bfd (control vs PTEN)	467.5	2459	1991	444.6	4.479	10	0,0011	##	Yes
SSs-AUD (control vs PTEN)	247.9	1421	1173	339.4	3.457	10	0,0061	##	Yes
Level 3									
RSP (control vs PTEN)	945.7	3358	2412	680.6	3.544	10	0,0053	##	Yes
VIS (control vs PTEN)	242.9	1253	1010	289.3	3.491	10	0,0058	##	Yes
AUD (control vs PTEN)	439.3	1651	1211	302.4	4.005	10	0,0024	##	Yes

PTEN vs TSC1*

Multiple t-test	Mean 1 (PTEN)	Mean 2 (TSC1)	Difference	SE of difference	t ratio	df	P value	Summary	Significant?
Level 1									
ACA (TSC1 vs PTEN)	3005	1656	1349	653.8	2.063	9	0,0691	*	Yes
MOs (TSC1 vs PTEN)	1084	371.4	712.2	295	2.414	9	0,0389	*	Yes
MOp (TSC1 vs PTEN)	821.9	356.3	465.6	276.3	1.685	9	0,1261	ns	No
SSp (TSC1 vs PTEN)	666.1	296.2	369.9	146.2	2.53	9	0,0322	*	Yes
Level 2									
RSP-MO (TSC1 vs PTEN)	2459	895.7	1563	517.6	3.02	9	0,0144	*	Yes
SSp (TSC1 vs PTEN)	1421	262.7	1159	381.6	3.036	9	0,0141	*	Yes
SSp-bfd (TSC1 vs PTEN)	1276	508.2	767.6	250.5	3.064	9	0,0134	*	Yes
SSs-AUD (TSC1 vs PTEN)	2057	467.8	1589	267.6	5.938	9	0,0002	***	Yes
Level 3									
RSP (TSC1 vs PTEN)	3358	1548	1809	791.4	2.286	9	0,048	*	Yes
VIS (TSC1 vs PTEN)	1253	691.9	561	324.2	1.731	9	0,1175	ns	No
AUD (TSC1 vs PTEN)	1651	900.1	750.5	338.1	2.22	9	0,0535	*	Yes

* Due to inequal variance (larger variation in pten mutants) we used mutiple-t-test to compare Pten data to either control ot Tsc1

ROSTRO-CAUDAL AXE

Quantification of CR fold changes in three neocortex levels : Control vs Mutants (Table 4.1)

	PIK3CA	TSC1	PTEN
	Mean ± SD	Mean ± SD	Mean ± SD
Level 1	2.6 ± 6.07	1.7 ± 2.33	3.64 ± 1.40
Level 2	4.1± 3.01	2.03± 7.59	6.65 ± 2.08
Level 3	2.6± 4.88	1.89 ± 4.20	3.63 ± 1.32

One sample t test	Theoretical mean	Actual mean	Discrepancy	95% CI of discrepancy	t. df	Std. Deviation	Std. Error of Mear	Lower 95% CI of mean	Upper 95% CI of mean	Sum	mber of valu	P value (two tailed)	Significant
Level 1													
PIK3CA	1	2.638	1.638	0.6722 to 2.605	t=5.396 df=3	0.6072	0.3036	1.672	3.605	10.55	4	0.0125	Yes
TSC1	1	1.747	0.7474	0.3764 to 1.118	t=6.412 df=3	0.2331	0.1166	1.376	2.118	6.99	4	0.0077	Yes
PTEN	1	3.645	2.645	1.347 to 3.942	t=4.986 df=6	1.403	0.5303	2.347	4.942	25.51	7	0.0025	Yes
Level 2													
PIK3CA	1	4.154	3.154	2.676 to 3.632	t=20.99 df=3	0.3006	0.1503	3.676	4.632	16.62	4	0.0002	Yes
TSC1	1	2.027	1.027	-0.1807 to 2.234	t=2.706 df=3	0.7588	0.3794	0.8193	3.234	8.107	4	0.0734	No
PTEN	1	6.652	5.652	3.732 to 7.571	t=7.204 df=6	2.076	0.7845	4.732	8.571	46.56	7	0.0004	Yes
Level 3													
PIK3CA	1	2.644	1.644	0.8672 to 2.421	t=6.734 df=3	0.4883	0.2442	1.867	3.421	10.58	4	0.0067	Yes
TSC1	1	1.894	0.8938	0.2254 to 1.562	t=4.255 df=3	0.42	0.21	1.225	2.562	7.575	4	0.0238	Yes
PTEN	1	3.633	2.633	1.414 to 3.852	t=5.285 df=6	1.318	0.4981	2.414	4.852	25.43	7	0.0019	Yes

Quantification of CR fold changes between levels in mutants

RM one way ANOVA table	SS	DF	MS	F (DFn, DFd)	P value	P value summary	ificant (alpha=0.05)?
PIK3CA							
Level	6.102	2	3.051	F (1.174, 3.521) = 21.24	0.0127	*	Yes
Matching	1.231	3	0.4103	F (3, 6) = 2.857	0.1268	ns	No
Residual (random)	0.8617	6	0.1436				
Total	8.195	11					
TSC1							
Level	0.1561	2	0.07806	F (1.643, 4.929) = 0.7027	0.5121	ns	No
Matching	1.753	3	0.5844	F (3, 6) = 5.26	0.0407	*	Yes
Residual (random)	0.6666	6	0.1111				
Total	2.576	11					
PTEN							
Level	42.37	2	21.18	F (1.229, 7.371) = 39.1	0.0002	***	Yes
Matching	41.58	6	6.931	F (6, 12) = 12.79	0.0001	***	Yes
Residual (random)	6.501	12	0.5418				
Total	90.45	20					

MEDIO-LATERAL AXE in three neocortex levels

Quantification of CR fold changes P24 _ Control vs Mutants (Table 4.2)

	PIK3CA	TSC1	PTEN
	Mean ± SD	Mean ± SD	Mean ± SD
Level 1			
ACA	1.3± 0.4	1.6 ± 0.30	3.0 ± 1.28
Mos	4.1 ± 1.08	1.0 ± 0.60	2.9 ± 1.50
Mop	5.3 ± 3.07	1.7 ± 1.26	4.0 ± 2.53
SSp	3.5 ± 0.79	2.84 ± 0.48	6.3 ± 2.72
Level 2			
RSP-MO	3.9± 0.29	1.9 ± 0.93	5.2 ± 2.06
SSp	4.8 ± 0.58	1.0 ± 0.53	5.8 ± 3.0
SSp-bfd	4.2 ± 0.78	3.3 ± 0.84	8.5 ± 3.20
SSs-AUD	3.6 ± 0.58	2.2 ± 0.85	10.0± 2.47
Level 3			
RSP	2.5 ± 0.76	1.6± 0.60	3.5 ± 1.57
VIS	4.4 ± 1.26	2.9 ± 0.50	5.1 ± 2.56
AUD	1.9 ± 0.5	2.0 ± 0.18	3.8 ± 1.50

Quantification of CR fold changes in the different areas of the level 1 P24 Control vs Mutants

One sample t test	Theoretical mean	Actual mean	Discrepancy	95% CI of discrepancy	t, df	Std. Deviation	Std. Error of Mear	Lower 95% CI of mean	Upper 95% CI of mean	Sum	mber of valu	P value (two tailed)	ificant (alpha=0.	value summa
ACA														
PIK3CA	1	1.377	0.3769	-0.3876 to 1.141	t=1.569, df=3	0.4805	0.2402	0.6124	2.141	5.508	4	0.2147	No	ns
TSC1	1	1.689	0.6894	0.1644 to 1.214	t=4.179, df=3	0.3299	0.1649	1.164	2.214	6.758	4	0.025	Yes	*
PTEN	1	3.066	2.066	0.8796 to 3.252	t=4.261, df=6	1.283	0.4848	1.88	4.252	21.46	7	0.0053	Yes	##
Mos														
PIK3CA	1	4.175	3.175	1.455 to 4.896	t=5.874, df=3	1.081	0.5405	2.455	5.896	16.7	4	0.0098	Yes	**
TSC1	1	1.006	0.006385	-0.9446 to 0.9574	t=0.02137, df=3	0.5977	0.2988	0.05539	1.957	4.026	4	0.9843	No	ns
PTEN	1	2.936	1.936	0.5454 to 3.327	t=3.406, df=6	1.504	0.5685	1.545	4.327	20.55	7	0.0144	Yes	#
Mop														
PIK3CA	1	5.366	4.366	-0.5213 to 9.254	t=2.843, df=3	3.071	1.536	0.4787	10.25	21.46	4	0.0655	No	ns
TSC1	1	1.771	0.771	-1.232 to 2.774	t=1.225, df=3	1.259	0.6295	-0.2324	3.774	7.084	4	0.3081	No	ns
PTEN	1	4.085	3.085	0.7445 to 5.427	t=3.225, df=6	2.531	0.9567	1.744	6.427	28.6	7	0.018	Yes	#
SSp														
PIK3CA	1	3.538	2.538	1.278 to 3.799	t=6.407, df=3	0.7924	0.3962	2.278	4.799	14.15	4	0.0077	Yes	**
TSC1	1	2.841	1.841	1.084 to 2.597	t=7.744, df=3	0.4754	0.2377	2.084	3.597	11.36	4	0.0045	Yes	**
PTEN	1	6.388	5.388	2.873 to 7.902	t=5.243, df=6	2.719	1.028	3.873	8.902	44.71	7	0.0019	Yes	##

Quantification of CR fold changes between areas of of level 1 in mutants

RM one way ANOVA table	SS	DF	MS	F (DFn, DFd)	P value	P value summary	ificant (alpha=0.05)?
PIK3CA							
Area	33.58	3	11.19	F (1,121, 3,364) = 5.416	0,0924	ns	No
Matching	15.78	3	5.261	F (3, 9) = 2.546	0,1213	ns	No
Residual (random)	18.6	9	2.067				
Total	67.96	15					
TSC1							
Area	6.893	3	2.298	F (1.257, 3.770) = 4.086	0.1169	ns	No
Matching	1.771	3	0.5903	F (3, 9) = 1.050	0.4169	ns	No
Residual (random)	5.061	9	0.5623				
Total	13.72	15					
PTEN							
Area	53.59	3	17.86	F (1.719, 10.31) = 5.743	0.0242	*	Yes
Matching	50.24	6	8.374	F (6, 18) = 2.692	0.0481	*	Yes
Residual (random)	55.99	18	3.11				
Total	159.8	27					

Quantification of CR fold changes in the different areas of the level 2 P24 Control vs Mutants

One sample t test	Theoretical mean	Actual mean	Discrepancy	95% CI of discrepancy	t, df	Std. Deviation	Std. Error of Mear	Lower 95% CI of mean	Upper 95% CI of mean	Sum	mber of valu	P value (two tailed)	ificant (alpha=0.	value summa
RSP-MO														
PIK3CA	1	4.807	3.807	2.896 to 4.718	t=13.30, df=3	0.2832	0.1416	3.54	4.441	15.96	4	0.0009	Yes	***
TSC1	1	1.06	0.05973	-0.7939 to 0.9134	t=0.2227, df=3	0.9306	0.4653	0.4353	3.397	7.664	4	0.8381	No	ns
PTEN	1	5.734	4.734	1.974 to 7.493	t=4.197, df=6	2.061	0.7789	3.354	7.166	36.82	7	0.0057	Yes	##
SSp														
PIK3CA	1	4.807	3.807	2.896 to 4.718	t=13.30, df=3	0.5725	0.2862	3.896	5.718	19.23	4	0.0009	Yes	***
TSC1	1	1.06	0.05973	-0.7939 to 0.9134	t=0.2227, df=3	0.5365	0.2682	0.2061	1.913	4.239	4	0.8381	No	ns
PTEN	1	5.734	4.734	1.974 to 7.493	t=4.197, df=6	2.984	1.128	2.974	8.493	40.14	7	0.0057	Yes	##
SSp-bfd														
PIK3CA	1	4.24	3.24	2.010 to 4.470	t=8.383, df=3	0.773	0.3865	3.01	5.47	16.96	4	0.0036	Yes	**
TSC1	1	3.385	2.385	1.033 to 3.736	t=5.617, df=3	0.8491	0.4245	2.033	4.736	13.54	4	0.0112	Yes	*
PTEN	1	8.497	7.497	4.533 to 10.46	t=6.190, df=6	3.204	1.211	5.533	11.46	59.48	7	0.0008	Yes	###
SSs-AUD														
PIK3CA	1	3.698	2.698	1.777 to 3.619	t=9.322, df=3	0.5789	0.2895	2.777	4.619	14.79	4	0.0026	Yes	**
TSC1	1	2.277	1.277	-0.07697 to 2.630	t=3.001, df=3	0.8507	0.4254	0.923	3.63	9.107	4	0.0576	No	ns
PTEN	1	10.01	9.01	6.723 to 11.30	t=9.640, df=6	2.473	0.9346	7.723	12.3	70.07	7	<0.0001	Yes	####

Quantification of CR fold changes between areas of of level 2 in mutants

RM one way ANOVA table	SS	DF	MS	F (DFn, DFd)	P value	P value summary	ificant (alpha=0.05)?
PIK3CA							
Area	2.66	3	0.8866	F (1.837, 5.511) = 3.410	0.1102	ns	No
Matching	1.682	3	0.5605	F (3, 9) = 2.156	0.1632	ns	No
Residual (random)	2.34	9	0.26				
Total	6.682	15					
TSC1							
Area	11.13	3	3.711	F (2.361, 7.084) = 16.28	0.0019	**	Yes
Matching	5.743	3	1.914	F (3, 9) = 8.398	0.0056	**	Yes
Residual (random)	2.052	9	0.228				
Total	18.93	15					
PTEN							
Area	107.6	3	35.87	F (2.004, 12.03) = 10.34	0.0024	**	Yes
Matching	114.7	6	19.12	F (6, 18) = 5.512	0.0022	**	Yes
Residual (random)	62.46	18	3.47				
Total	284.8	27					

Quantification of CR fold changes in the different areas of the level 3 P24 Control vs Mutants

One sample t test	Theoretical mean	Actual mean	Discrepancy	95% CI of discrepancy	t, df	Std. Deviation	Std. Error of Mear	Lower 95% CI of mean	Upper 95% CI of mean	Sum	mber of valu	P value (two tailed)	ificant (alpha=0.	value summa
RSP														
PIK3CA	1	4.403	3.403	1.398 to 5.408	t=5.402, df=3	0.7687	0.3844	1.268	3.714	9.965	4	0.0124	Yes	*
TSC1	1	2.849	1.849	1.045 to 2.653	t=7.318, df=3	0.64	0.32	0.6189	2.656	6.549	4	0.0053	Yes	**
PTEN	1	5.158	4.158	1.769 to 6.548	t=4.259, df=6	1.571	0.5939	2.097	5.003	24.85	7	0.0053	Yes	##
VIS														
PIK3CA	1	4.403	3.403	1.398 to 5.408	t=5.402, df=3	1.26	0.63	2.398	6.408	17.61	4	0.0124	Yes	*
TSC1	1	2.849	1.849	1.045 to 2.653	t=7.318, df=3	0.5052	0.2526	2.045	3.653	11.39	4	0.0053	Yes	**
PTEN	1	5.158	4.158	1.769 to 6.548	t=4.259, df=6	2.583	0.9764	2.769	7.548	36.11	7	0.0053	Yes	##
AUD														
PIK3CA	1	1.838	0.838	0.02454 to 1.651	t=3.278, df=3	0.5112	0.2556	1.025	2.651	7.352	4	0.0465	Yes	*
TSC1	1	2.049	1.049	0.7625 to 1.335	t=11.65, df=3	0.18	0.09001	1.763	2.335	8.196	4	0.0014	Yes	**
PTEN	1	3.757	2.757	1.371 to 4.143	t=4.868, df=6	1.498	0.5664	2.371	5.143	26.3	7	0.0028	Yes	##

Quantification of CR fold changes between areas of of level 3 in mutants

RM one way ANOVA table	SS	DF	MS	F (DFn, DFd)	P value	P value summary	ificant (alpha=0.05)?
PIK3CA							
Area	14.22	2	7.108	F (1.167, 3.501) = 11.33	0.0333	*	Yes
Matching	3.554	3	1.185	F (3, 6) = 1.888	0.2326	ns	No
Residual (random)	3.765	6	0.6276				
Total	21.54	11					
TSC1							
Area	3.035	2	1.518	F (1.124, 3.373) = 8.073	0.0563	ns	No
Matching	0.9639	3	0.3213	F (3, 6) = 1.709	0.2637	ns	No
Residual (random)	1.128	6	0.188				
Total	5.127	11					
PTEN							
Area	10.71	2	5.357	F (1.447, 8.679) = 2.973	0.1127	ns	No
Matching	46.7	6	7.784	F (6, 12) = 4.320	0.0150	*	Yes
Residual (random)	21.62	12	1.802				
Total	79.04	20					

Quantification of cortical layer thickness in the S1bfd (Table 4.3) of mutants and control at P24 (Table S4.3)

	Control (n=4)	PIK3CA (n=4)	TSC1 (n=4)	PTEN (n=5)
	Mean ± SD	Mean ± SD	Mean ± SD	Mean ± SD
Layer 1	92.9± 9.8	124.3± 14.8	91.8± 9.8	88.2 ±8.5
Layer 2/3	351.9± 39.9	318.3± 33.9	332.1± 32.8	308.4± 18.8
Layer 4	179.5± 20.5	166.8± 16.7	160.9± 22.1	172.8 ±9.7
Layer 5	310.2± 24.1	270.3± 37.3	272.4± 24.4	285.7 ±12.1
Layer 6	328.4± 29.0	341.7± 57.6	345.8± 34.2	319.8 ±29.7

Multiple t-test PIK3CA vs. Ct	P value	Mean of PIK3CA	Mean of Control	Difference	SE of difference	t ratio	df	q value
Layer 1	**0,012558**	124,3	92,99	31,28	8,893	3,517	6	0,06342
Layer 2/3	0,247213	318,3	351,9	-33,56	26,18	1,282	6	0,416141
Layer 4	0,371403	166,8	179,6	-12,8	13,25	0,9659	6	0,468896
Layer 5	0,122642	270,3	310,2	-39,95	22,25	1,796	6	0,309672
Layer 6	0,696166	341,7	328,4	13,22	32,27	0,4098	6	0,703127

Multiple t-test TSC1 vs. Ct	P value	Mean of TSC1	Mean of Control	Difference	SE of difference	t ratio	df	q value
Layer 1	0,875045	91,85	92,99	-1,137	6,93	0,1641	6	0,883795
Layer 2/3	0,47313	332,1	351,9	-19,76	25,82	0,7653	6	0,597326
Layer 4	0,263559	161	179,6	-18,63	15,1	1,233	6	0,597326
Layer 5	0,069817	272,4	310,2	-37,82	17,17	2,203	6	0,352573
Layer 6	0,468796	345,8	328,4	17,35	22,44	0,7731	6	0,597326

Multiple t-test PTEN vs. Ct	P value	Mean of PTEN	Mean of Control	Difference	SE of difference	t ratio	df	q value
Layer 1	0,465568	88,27	92,99	-4,721	6,117	0,7717	7	0,677928
Layer 2/3	0,065961	308,5	351,9	-43,42	19,95	2,177	7	0,21681
Layer 4	0,536973	172,9	179,6	-6,682	10,29	0,6491	7	0,677928
Layer 5	0,085866	285,7	310,2	-24,51	12,27	1,998	7	0,21681
Layer 6	0,676575	319,9	328,4	-8,588	19,74	0,4351	7	0,683341

Quantification of tdT positive CR volume (Table 5.1)

	Control	PTEN
	Mean ± SD	Mean ± SD
volume (control vs PTEN)	382.5± 221.5	1144.5±644.4

Mann-Whitney U test	*Two-tailed p* value	U	Significant (alpha=0.05)?	P value summary
volume (control vs PTEN)	<0.0001	128	Yes	****

Quantification of Reelin intensity in tdT positive CRs (Table 5.2)

	Control	PTEN
	Mean ± SD	Mean ± SD
RLEN intensity (control vs PTEN)	176± 93.87	348.8 ±246.3

Mann-Whitney U test	*Two-tailed p* value	U	Significant (alpha=0.05)?	P value summary
RLEN intensity (control vs PTEN)	0,0026	442	Yes	**

Quantification of CR intrinsic properties in the layer one above the SSp-bfd (Table 6.1)

Mean ± SEM

Intrinsic Properties	Control (n=14)	PTEN (n=12)	paired t -test (p value)
Vm (mV)	-50.3 ± 0.43	-46.7 ± 0.88	0.0095
Rm (MOhms)	1290.7 ± 98.8	745.65 ± 152.5	0.0379
Cm (pF)	21.15 ± 1.06	48.34 ± 2.23	< 0.0001

Quantification of CR Action potentials in the layer one above the SSp-bfd (Table 6.2)

Action Potentials (AP)

Frequency	Control (n=14)	PTEN (n=12)	anova 2 ways
10	0 ± 0	0 ± 0	>0.9999
20	1.7± 1.28	0.42 ± 0.42	>0.9999
30	5.8 ± 2.30	2 ± 1.17	0.9788
40	8.9 ± 2.53	7 ± 1.70	>0.9999
50	9.36 ± 2.15	11.4 ± 2.11	>0.9999
60	10 ± 2.41	15.4 ± 2.51	0.9105
70	11.4 ± 3.13	20.2 ± 2.32	0.2774
80	12.4 ± 3.37	23.3 ± 2.35	0.0427
90	13.5 ± 4.08	27.1 ± 2.46	0.0069
100	15.4 ± 5.07	29.7 ± 2.49	0.0039
110	15.2± 5.74	32.8 ± 2.31	0.001
120	17.1 ± 5.24	34.3 ± 1.91	0.0002

Amplitude (mV)	Control (n=14)	PTEN (n=12)	anova 2 ways
1	1.2 ± 2.22	77.3 ± 1.73	P < 0.0001
2	44.9 ± 1.84	74.6 ± 2.03	P < 0.0001
3	41.7 ± 2.79	73.5 ± 2.08	P < 0.0001
4	40.0 ± 2.45	72.4 ± 2.38	P < 0.0001
5	36.5 ± 2.56	71.9 ± 2.47	P < 0.0001

	Control (n=14)	PTEN (n=12)	paired t -test (p value)
Threshold (mV)	-41.05 ± 5.47	-44.505 ± 1.2	0.0434
Halfwidth (ms)	2.65 ± 0.11	1.53 ± 0.05	0.0001
Latency (ms)	98.138 ± 23.6	246.2 ± 36.3	0.0128

Quantification of the extra-cellular reelin signal intensity in layer 1 of the future S1 cortex in PTEN cKO and control mice at P1 (Table 7.1)

	Control	PTEN
Mean	229,9	354,1
SD	133,6	213
n (4 mice*4 ROI/layerI)	16	16

Mann Whitney test	
P value	0,0938
Exact or approximate P value?	Exact
P value summary	ns
Significantly different (P < 0.05)?	No
One- or two-tailed P value?	Two-tailed
Sum of ranks in column A,B	219 , 309
Mann-Whitney U	83
Difference between medians	
Median of Control	194.2, n=16
Median of PTEN	262.9, n=16
Difference: Actual	68,65
Difference: Hodges-Lehmann	94,12

Quantification of the reelin expression and cleavage in cortex from PTEN cKO and control mice at P7-P8 (Table 7.2)

**Reelin Expression** (FL+N-R6+N-R2)/Pan-Cadherin	
Unpaired t test	
P value	0,43
P value summary	ns
Significantly different (P < 0.05)?	No
One- or two-tailed P value?	Two-tailed
t, df	t=0.8459, df=6
How big is the difference?	
Mean of Control	1,425
Mean of PTEN	1,615
Difference between means (B - A) ± SEM	0.1900 ± 0.2246
95% confidence interval	-0.3596 to 0.7396
R squared (eta squared)	0,1066
F test to compare variances	
F, DFn, Dfd	1.682, 3, 3
P value	0,6796
P value summary	ns
Significantly different (P < 0.05)?	No
Data analyzed	
Sample size, control	4
Sample size, PTEN	4

**Reelin cleavage** (N-R6+N-R2)/(FL+N-R6+N-R2)	
Unpaired t test	
P value	**0,0355**
P value summary	*
Significantly different (P < 0.05)?	Yes
One- or two-tailed P value?	Two-tailed
t, df	t=2.702, df=6
How big is the difference?	
Mean of control	0,9975
Mean of PTEN	0,86
Difference between means (B - A) ± SEM	-0.1375 ± 0.05089
95% confidence interval	-0.2620 to -0.01298
R squared (eta squared)	0,5489
F test to compare variances	
F, DFn, Dfd	1.967, 3, 3
P value	0,5926
P value summary	ns
Significantly different (P < 0.05)?	No
Data analyzed	
Sample size, control	4
Sample size, PTEN	4

Quantification of the pS6 signal intensity in cortex from PTEN cKO and control mice at P1 (Table 7.3)

Two-way ANOVA	Ordinary				
Alpha	0,05				
Source of Variation	% of total variation	P value	P value summary	Significant?	
Interaction	11,59	0,266	ns	No	
bin	28,95	0,0033	**	Yes	
genotype	2,249	0,1398	ns	No	
ANOVA table	SS (Type III)	DF	MS	F (DFn, DFd)	P value
Interaction	289753	9	32195	F (9, 57) = 1.283	P=0.2660
bin	724038	9	80449	F (9, 57) = 3.207	P=0.0033
genotype	56238	1	56238	F (1, 57) = 2.242	P=0.1398
Residual	1429844	57	25085		
Difference between column means					
Predicted (LS) mean of Lima	200				
Predicted (LS) mean of Rolling-Stones	254,3				
Difference between predicted means	-54,34				
SE of difference	36,29				
95% CI of difference	-127.0 to 18.33				
Normality of Residuals					
Test name	Statistics	P value	Passed normality test (alpha	P value summary	
Anderson-Darling (A2*)	0,6291	0,0976	Yes	ns	
D'Agostino-Pearson omnibus (K2)	1,396	0,4977	Yes	ns	
Shapiro-Wilk (W)	0,9716	0,0821	Yes	ns	
Kolmogorov-Smirnov (distance)	0,07658	0,1	Yes	ns	

Quantification of the burst and seizures neuronal network activity in S1 cortex in adult PTEN cKO and control mice (Table 7.4)

Type of activity				
Fisher's exact test	*p* value	odds ratio	Significant (alpha=0.05)?	P value summary
two-sided	1	0,9231	No	ns

Burst properties							
unpaired t-test	*Two-tailed p* value	N (CTR)	N (PTEN)	(CTR) Mean ± SEM	(PTEN) Mean ± SEM	Significant (alpha=0.05)?	P value summary
burst frequency	0.9739	13	14	4.735 ± 0.7335	4.776 ± 0.9932	No	ns
burst duration	0.5072	13	14	1.651 ± 0.1791	1.854 ± 0.2398	No	ns

Behavior analysis of adult PTEN cKO and control mice (Table 8.1)

Y-Maze Spontaneous Alternation

D'Agostino & Pearson normality test	K2	P value	Passed normality test (alpha=0	P value summary
Control Female	0.8746	0.6458	Yes	ns
PTEN Female	3.64	0.162	Yes	ns
Control Male	0.3284	0.8486	Yes	ns
PTEN Male	1.706	0.426	Yes	ns

One sample t test	Theoretical mean	Actual mean	Discrepancy	5% CI of discrepanc	t. df	P value (two tailed)	Significant (alpha=0.05)?
Control Female	50	60.8	10.8	7.398 to 14.19	t=6.628. df=20	<0.0001	Yes
PTEN Female	50	63.69	13.69	8.162 to 19.22	t=5.251. df=16	<0.0001	Yes
Control Male	50	61.13	11.13	6.126 to 16.13	t=4.742. df=15	0.0003	Yes
PTEN Male	50	60.84	10.84	6.972 to 14.72	t=5.937. df=16	<0.0001	Yes

Vertical activity

Two ways ANOVA table	SS (Type III)	DF	MS	F (DFn, DFd)	P value	P value summary	Significant?
Rearing 10 minutes							
Interaction	636	1	636	F (1, 59) = 0.6630	0.4188	ns	No
sex	216.9	1	216.9	F (1, 59) = 0.2261	0.6362	ns	No
genotype	3798	1	3798	F (1, 59) = 3.960	0.0512	ns	No
Residual	56595	59	959.2				
Rearing 50 minutes							
Interaction	4029	1	4029	F (1, 59) = 0.1807	0.6723	ns	No
sex	7668	1	7668	F (1, 59) = 0.3439	0.5598	ns	No
genotype	110553	1	110553	F (1, 59) = 4.958	0.0298	*	Yes
Residual	1315485	59	22296				

Sidak's multiple comparisons test	Mean 1 (Control)	Mean 2 (PTEN)	Mean Diff.	SE of diff.	95.00% CI of diff.	N1 (Control)	N2 (PTEN)	t	DF	justed P Val	Summary	Significant?
Rearing 10 minutes												
Female Control vs PTEN	98.27	76.35	21.91	10.97	-3.264 to 47.09	15	17	1.997	59	0.0983	ns	No
Male Control vs PTEN	88.19	79	9.188	11.13	-16.36 to 34.73	16	15	0.8254	59	0.6548	ns	No
Rearing 10 minutes												
Female Control vs PTEN	399.7	299.8	99.91	52.9	-21.47 to 221.3	15	17	1.889	59	0.1236	ns	No
Male Control vs PTEN	405.8	337.9	67.88	53.67	-55.27 to 191	16	15	1.265	59	0.3773	ns	No

Horizontal Activity

Multiple t-test	Mean 1 (Control)	Mean 2 (PTEN)	Difference	SE of difference	t ratio	df	P value	Summary	Significant?	
Rearing Total Activity 10 minutes										
Female	3334	2768	566.4	327.6	1.729	35	0,0926	ns	No	
Male	2632	2266	366	324.5	1.128	29	0,2685	ns	No	

Rearing Total Activity 50 minutes

Two way anova ANOVA table	SS (Type III)	DF	MS	F (DFn, DFd)	P value	P value summary	Significant?
Interaction	16947	1	16947	F (1, 64) = 0.002143	0.9632	ns	No
Sex	14897559	1	14897559	F (1, 64) = 1.884	0.1747	ns	No
genotype	26471199	1	26471199	F (1, 64) = 3.347	0.0720	ns	No
Residual	506177882	64	7909029				

Open field (OF)

OF Entry center

Two way anova ANOVA table	SS (Type III)	DF	MS	F (DFn, DFd)	P value	P value summary	Significant?
Entry center 10min							
Interaction	16.49	1	16.49	F (1, 64) = 0.08432	0.7725	ns	No
Gender	48.45	1	48.45	F (1, 64) = 0.2478	0.6203	ns	No
Genotype	440.1	1	440.1	F (1, 64) = 2.251	0.1385	ns	No
Residual	12514	64	195.5				
Entry center 30 min							
Interaction	46.78	1	46.78	F (1, 64) = 0.05762	0.8111	ns	No
Gender	1593	1	1593	F (1, 64) = 1.962	0.1661	ns	No
Genotype	671.2	1	671.2	F (1, 64) = 0.8267	0.3666	ns	No
Residual	51957	64	811.8				
Entry center 1 h							
Interaction	916.2	1	916.2	F (1, 64) = 0.3509	0,5557	ns	No
Gender	2793	1	2793	F (1, 64) = 1.070	0,3048	ns	No
Genotype	8166	1	8166	F (1, 64) = 3.128	0,0817	ns	No
Residual	167075	64	2611				

OF Total distance

Two way anova ANOVA table	SS (Type III)	DF	MS	F (DFn, DFd)	P value	P value summary	Significant?
OF Total distance 10min							
Interaction	618196	1	618196	F (1, 65) = 0.3654	0.5476	ns	No
Gender	6834416	1	6834416	F (1, 65) = 4.040	0.0486	*	Yes
Genotype	566385	1	566385	F (1, 65) = 0.3348	0.5648	ns	No
Residual	109960040	65	1691693				
OF Total distance 30 min							
Interaction	3908881	1	3908881	F (1, 65) = 0.613	0.4365	ns	No
Gender	6979313	1	6979313	F (1, 65) = 1.094	0.2994	ns	No
Genotype	527564	1	527564	F (1, 65) = 0.08273	0.7745	ns	No
Residual	414506258	65	6377019				
OF Total distance 1 h							
Interaction	17653	1	17653	F (1, 64) = 0.001169	0.9728	ns	No
Gender	4229173	1	4229173	F (1, 64) = 0.2800	0.5985	ns	No
Genotype	41825240	1	41825240	F (1, 64) = 2.769	0.1010	ns	No
Residual	966690114	64	15104533				

OF Time in the center

Two way anova ANOVA table	SS (Type III)	DF	MS	F (DFn, DFd)	P value	P value summary	Significant?
OF Time center 10 min							
Interaction	63,08	1	63,08	F (1, 63) = 0.1705	0.6811	ns	No
Gender	40,62	1	40,62	F (1, 63) = 0.1098	0.7415	ns	No
Genotype	1556	1	1556	F (1, 63) = 4.205	0.0445	*	Yes
Residual	23310	63	370				
OF Time center 30 min							
Interaction	395,3	1	395,3	F (1, 65) = 0.08740	0.7685	ns	No
Gender	3738	1	3738	F (1, 65) = 0.8263	0.3667	ns	No
Genotype	3772	1	3772	F (1, 65) = 0.8339	0.3645	ns	No
Residual	293999	65	4523				
OF Time center 1h min							
Interaction	4576	1	4576	F (1, 64) = 0.3073	0.5813	ns	No
Gender	26821	1	26821	F (1, 64) = 1.801	0.1843	ns	No
Genotype	10887	1	10887	F (1, 64) = 0.7311	0.3957	ns	No
Residual	953003	64	14891				

Sidak's multiple comparisons test	Mean 1 F	Mean 2 M	Mean Diff.	SE of diff.	95.00% CI of diff.	N1	N2	t	DF	justed P Val	Summary	Significant?
Behavior OF Time center 10 min												
PTEN	46,11	49,62	-3,511	6,814	-19.12 to 12.10	17	15	0,5153	63	0,8465	ns	No
Control	57,73	57,35	0,3847	6,527	-14.57 to 15.34	19	16	0,05895	63	0,9978	ns	No

OF ratio Center to total distance

Two way anova ANOVA table	SS (Type III)	DF	MS	F (DFn, DFd)	P value	P value summary	Significant?
OF ratio 10 min							
Interaction	0.00003316	1	0.00003316	F (1, 64) = 0.02689	0.8703	ns	No
Gender	0.000005945	1	0.000005945	F (1, 64) = 0.004821	0.9449	ns	No
Genotype	0.007357	1	0.007357	F (1, 64) = 5.966	0.0174	*	Yes
Residual	0.07892	64	0.001233				
OF ratio 30 min							
Interaction	0.00001148	1	0.00001148	F (1, 65) = 0.01041	0.9191	ns	No
Gender	0.001375	1	0.001375	F (1, 65) = 1.247	0.2682	ns	No
Genotype	0.001847	1	0.001847	F (1, 65) = 1.675	0.2001	ns	No
Residual	0.07168	65	0.001103				
OF ratio 1h min							
Interaction	0.00001209	1	0.00001209	F (1, 65) = 0.01495	0.9030	ns	No
Gender	0.0005724	1	0.0005724	F (1, 65) = 0.7078	0.4033	ns	No
Genotype	0.003107	1	0.003107	F (1, 65) = 3.843	0.0543	ns	No
Residual	0.05257	65	0.0008087				

Sidak's multiple comparisons test	Mean 1 F	Mean 2 M	Mean Diff.	SE of diff.	95.00% CI of diff.	N1	N2	t	DF	usted P Va	Summary	Significant?
Behavior OF ratio 10 min												
PTEN	0.132	0.13	0.001999	0.01244	-0.02649 to 0.03049	17	15	0.1607	64	0.9838	ns	No
Control	0.1515	0.1523	-0.00081	0.01178	-0.02778 to 0.02616	20	16	0.06877	64	0.997	ns	No

Sensitivity of adult PTEN or pik3CA mutants to kainate induced seizures compared to controls (Table 8.2)

PTEN

Mann-Whitney U test	*Two-tailed p* value	U	(Control) Mean ± SD	N (Control)	(PTEN) Mean ± SD	N (PTEN)	Significant (alpha=0.05)?	P value summary
latency (min)								
Females	0,7835	16	26.2 ± 46.4	6	15.5 ± 27.2	6	No	ns
Males	0,9697	15 1/2	36.6 ±48.8	4	35.5± 49.9	8	No	ns
Duration (min)								
Females	**0,0455**	5,5	35.7 ± 44.1	6	87.6 ± 33.2	6	Yes	*
Males	0,802	14	18.7 ± 18.6	4	13.6 ± 13	8	No	ns
Number of occurence								
Females	0,4459	12,5	1.3 ± 1.0	6	2.2 ± 1.6	6	No	ns
Males	0,6687	13	0.7 ± 0.5	4	1.0 ± 0.7	8	No	ns

PIK3CA

Mann-Whitney U test	*Two-tailed p* value	U	(Control) Mean ± SD	N (Control)	(PIK3CA) Mean ± SD	N (PIK3CA)	Significant (alpha=0.05)?	P value summary
latency (min)								
Females	0.753	24	17 ± 9.17	6	17.8 ± 9.34	9	No	ns
Males	0.7902	22	86.0 ± 4.80 (10)	8	89.5 ± 4.73(10)	6	No	ns
Duration (min)								
Females	**0.0076**	5	28.2± 2.51(10)	6	75.2 ± 3.04 (10)	9	Yes	**
Males	0.7902	22	6.5 ± 1.09 (10)	8	17.05 ± 2.65 (10)	6	No	ns
Number of occurance								
Females	0.3794	19,5	1.8 ± 0.98	6	2.8 ± 2.02	9	No	ns
Males	0.8741	22,5	2.1 ± 3.22	8	2.8 ± 4.40	6	No	ns

Sensitivity of adult PTEN or pik3CA mutants to kainate induced seizures compared to controls (Table 8.2)

PTEN

Mann-Whitney U test	*Two-tailed p* value	U	(Control) Mean ± SD	N (Control)	(PTEN) Mean ± SD	N (PTEN)	Significant (alpha=0.05)?	P value summary
latency (min)								
Females	0,7835	16	26.2 ± 46.4	6	15.5 ± 27.2	6	No	ns
Males	0,9697	15 1/2	36.6 ±48.8	4	35.5± 49.9	8	No	ns
Duration (min)								
Females	**0,0455**	5,5	35.7 ± 44.1	6	87.6 ± 33.2	6	Yes	*
Males	0,802	14	18.7 ± 18.6	4	13.6 ± 13	8	No	ns
Number of occurence								
Females	0,4459	12,5	1.3 ± 1.0	6	2.2 ± 1.6	6	No	ns
Males	0,6687	13	0.7 ± 0.5	4	1.0 ± 0.7	8	No	ns

PIK3CA

Mann-Whitney U test	*Two-tailed p* value	U	(Control) Mean ± SD	N (Control)	(PIK3CA) Mean ± SD	N (PIK3CA)	Significant (alpha=0.05)?	P value summary
latency (min)								
Females	0.753	24	17 ± 9.17	6	17.8 ± 9.34	9	No	ns
Males	0.7902	22	86.0 ± 4.80 (10)	8	89.5 ± 4.73(10)	6	No	ns
Duration (min)								
Females	**0.0076**	5	28.2± 2.51(10)	6	75.2 ± 3.04 (10)	9	Yes	**
Males	0.7902	22	6.5 ± 1.09 (10)	8	17.05 ± 2.65 (10)	6	No	ns
Number of occurance								
Females	0.3794	19,5	1.8 ± 0.98	6	2.8 ± 2.02	9	No	ns
Males	0.8741	22,5	2.1 ± 3.22	8	2.8 ± 4.40	6	No	ns

Table S9: Supplemental Material and Methods- list of reagents and softwares

Reagent type (species) or resource	Designation	Source or reference	Identifiers	Additional information
**Strain Mus musculus (males and female)**	C57BL6J	Janvier		
**Mus musculus (males and female)**	$\Delta Np73^{CreIRESGFP}$	[20]	$\Delta Np73^{Cre}$	
**Mus musculus (males and female)**	$ROSA26^{loxP\text{-}stop\text{-}loxP\text{-}Tomato}$	[38]	$R26^{mT}$	
**Mus musculus (males and female)**	*R26StopFlp110**	[24]	$PIK3CA^{p110*}$	
**Mus musculus (males and female)**	$Pten^{loxP}$	[39]	$PTEN^{lox/lox}$	
**Mus musculus (males and female)**	$TSC1^{loxP}$	[40]	$TSC1^{del/lox}$	

**Sequence-based reagent**	ΔNp73$^{Cre/+}$ genotyping<br>delta1<br>delta2<br>83RC	This Manuscript	PCR primers	delta1:<br>GAA-TGC-CAA-CTC-TCA-GTC-CG<br>delta2:<br>GTC-TCT-CTG-AAC-CCC-AAC-CA<br>83RC:<br>GGT-GTA-CGG-GCA-GTA-AAT-TGG-AC
**Sequence-based reagent**	R26^{mT} genotyping<br>RCE-1<br>RCE-2<br>RCE-3	This Manuscript	PCR primers	RCE-1:<br>CCC-AAA-GTC-GCT-CTG-AGT-TGT-TAT-C<br>RCE-2:<br>GAA-GGA-GCG-GGA-GAA-ATG-GAT-ATG<br>RCE-3:<br>CCA-GGC-GGG-CCA-TTT-ACC-GTA-AG
**Sequence-based reagent**	Rosap110* genotyping<br>RCE-1<br>RCE-2<br>NeoR1	This Manuscript	PCR primers	RCE-1:<br>CCC-AAA-GTC-GCT-CTG-AGT-TGT-TAT-C<br>RCE-2:<br>GAA-GGA-GCG-GGA-GAA-ATG-GAT-ATG

				NeoR1:<br><br>GAC-ATC-ATC-AAG-GAA-ACC-CTG-GAC
**Sequence-based reagent**	PTEN genotyping New_Sens<br><br>Del_AS<br><br>i5_S	[39]	PCR primers	New_Sens:<br><br>AGA-TTG-TAT-GTG-ATC-ATC-TGT-CAG-G<br><br>Del_AS:<br><br>AGC-AGG-TGA-AAG-AGA-AGT-ACA-G<br><br>i5_S:<br><br>ATT-AAA-TTT-GGA-CTT-GTG-CCC-CA
**Sequence-based reagent**	TSC1 genotyping<br><br>F4536<br><br>R4830<br><br>R6548	[40]	PCR primers	F4536:<br><br>AGG-AGG-CCT-CTT-CTG-CTA-CC<br><br>R4830:<br><br>CAG-CTC-CGA-CCA-TGA-AGT-G<br><br>R6548:<br><br>TGG-GTC-CTG-ACC-TAT-CTC-CTA
**Antibody**	goat polyclonal IgG anti-reelin antibody	R&D Systems, USA	Cat# AF3820,<br><br>RRID:AB_2253745	IF(1:500)

**Antibody**	mouse monoclonal IgG1 anti-reelin antibody (G10)	Millipore (Merck), USA	Cat# MAB5364, RRID: AB_11212203	WB (1:500)
**Antibody**	mouse monoclonal anti-panCadherin antibody (CH-19)	Sigma-Aldrich, USA	Cat# C1821, RRID: AB_476826	WB (1:1000)
**Antibody**	rabbit monoclonal anti-phospho-AKT Serine 473 antibody (D9E)	Cell signaling Technology, USA	Cat# 4060, RRID:AB_2315049	IF(1:200)
**Antibody**	rabbit monoclonal anti-phospho-S6 antibody (Ser 240/244) (D68F8)	Cell signaling Technology, USA	Cat# 5364, RRID:AB_10694233	IF(1:1000)
**Antibody**	rat monoclonal anti-ctip2 antibody	Abcam, USA	Cat# ab18465, RRID:AB_2064130	IF(1:250)
**Antibody**	rabbit polyclonal anti-cux1,2 antibody	Santa Cruz Biotechnology, USA	Cat# sc13024, RRID:AB_2261231	IF(1:250)

**Antibody**	donkey anti-rabbit Alexa-488	Jackson ImmunoResearch Laboratories, USA	Cat# 711-545-152,<br>RRID: AB_2313584	IF(1:500)
**Antibody**	donkey anti-goat Alexa-488	Molecular Probes, USA	Cat# A11055,<br>RRID:AB_2534102	IF(1:500)
**Antibody**	donkey anti-goat Cy5	Jackson ImmunoResearch Laboratories, USA	Cat# 705-175-147,<br>RRID: AB_2340415	IF(1:250)
**Antibody**	donkey anti-rat Alexa-Cy5	Jackson ImmunoResearch Laboratories, USA	Cat# 712-175-153,<br>RRID: AB_2340672	IF(1:250)
**Antibody**	Polyclonal anti-mouse IgG, Fc<br>HRP	Jackson ImmunoResearch Laboratories, USA	Cat# 115-035-008,<br>RRID: AB_2313585	WB(1:20 000)

**Antibody**	Zenon® Alexa Fluor® 488 Rabbit IgG Labeling Kit	Thermo Fisher Scientific, USA	Cat# Z25302, RRID:AB_2572214	
**Antibody**	DAPI (4',6-Diamidino-2-Phenylindole, Dihydrochloride)	ThermoFisher Scientific, USA	Cat# D1306, RRID:AB_2629482	IF(1:1000)
**Antibody**	Biocytin	ThermoFisher Scientific, USA	Cat#B1592	
**Antibody**	Streptavidin, Alexa Fluor™ 546 conjugate	Thermo Fisher Scientific, USA	Cat#S11225 RRID:AB_2532130	
**Chemical compound, drug**	Vectashield	Vector Laboratories, USA	Cat# H1000, RRID:AB_2336789	
**Chemical compound, drug**	Normal Goat Serum (10%)	Thermo Fisher Scientific, USA	cat # 50062Z	

**Chemical compound, drug**	ProLong™ Diamond Antifade Mountant	Thermo Fisher Scientific, USA	cat # P36970	
**Chemical compound, drug**	Triton 100X	Eurobio, France	Cat# GAUTTR00-07	
**Chemical compound, drug**	Paraformaldehyde	Sigma-Aldrich, USA	Cat# P6148	
**Chemical compound, drug**	Tissue Tek * OCT compound	Sakura Finetek, USA	Cat# NC1862249	
**Chemical compound, drug**	Gelatin from porcine skin	Sigma-Aldrich, USA	Cat# G2500	
**Chemical compound, drug**	Ketamine (Imalgen 1000)	Centravet, Dinan, France	GTIN: 03661103003199	180mg/kg
**Chemical compound, drug**	Xylasine (Rompun 2%)	Centravet, Dinan, France	GTIN: 04007221032311	10mg/kg

**Chemical compound, drug**	Kainate	Bio-Techne, USA	Cat#0222/10	15mg/Kg
**Software, algorithm**	ImageJ/FIJI	National Institutes of Health (NIH), USA	RRID:SCR_002285	
**Software, algorithm**	Adobe Photoshop CS6	Adobe Inc., USA	RRID:SCR_014199	
**Software, algorithm**	GraphPad Prism 8.0	GraphPad Software, USA	RRID:SCR_000306	
**Software, algorithm**	CorelDraw Graphics Suite	Corel Corporation, Canada	RRID:SCR_014235	
**Software, algorithm**	Axon™ pCLAMP® 9 Software	Molecular Devices, USA	RRID:SCR_011323	
**Software, algorithm**	IGORpro	WaveMetrics Inc, USA	RRID:SCR_000325	
**Software, algorithm**	TaroTools	Jikei University School of		

		Medicine, Japan		
**Software, algorithm**	Axograph software	Axograph Inc, USA	RRID:SCR_014284	
**Software, algorithm**	MEA Monitor software	Multichannel Systems, Germany		
**Software, algorithm**	MC Rack 4.5.1 software	Multichannel Systems, Germany	RRID:SCR_014955	
**Software, algorithm**	Videotrack v2.6 Automated Behavioural Analysis	ViewPoint, France		
**Software, algorithm**	Image Lab Software, V6.1	Biorad, France		
