# Supplementary material for: Activation of the PI3K/AKT/mTOR Pathway in Cajal–Retzius Cells Leads to Their Survival and Increases Susceptibility to Kainate-Induced Seizures
Source: Int J Mol Sci. 2023 Mar 11;24(6):5376. doi: 10.3390/ijms24065376 (PMC10048971; doi:10.3390/ijms24065376)
Supplement: Supplementary file 1 [file ijms-24-05376-s001.zip › Figure S1.pdf]

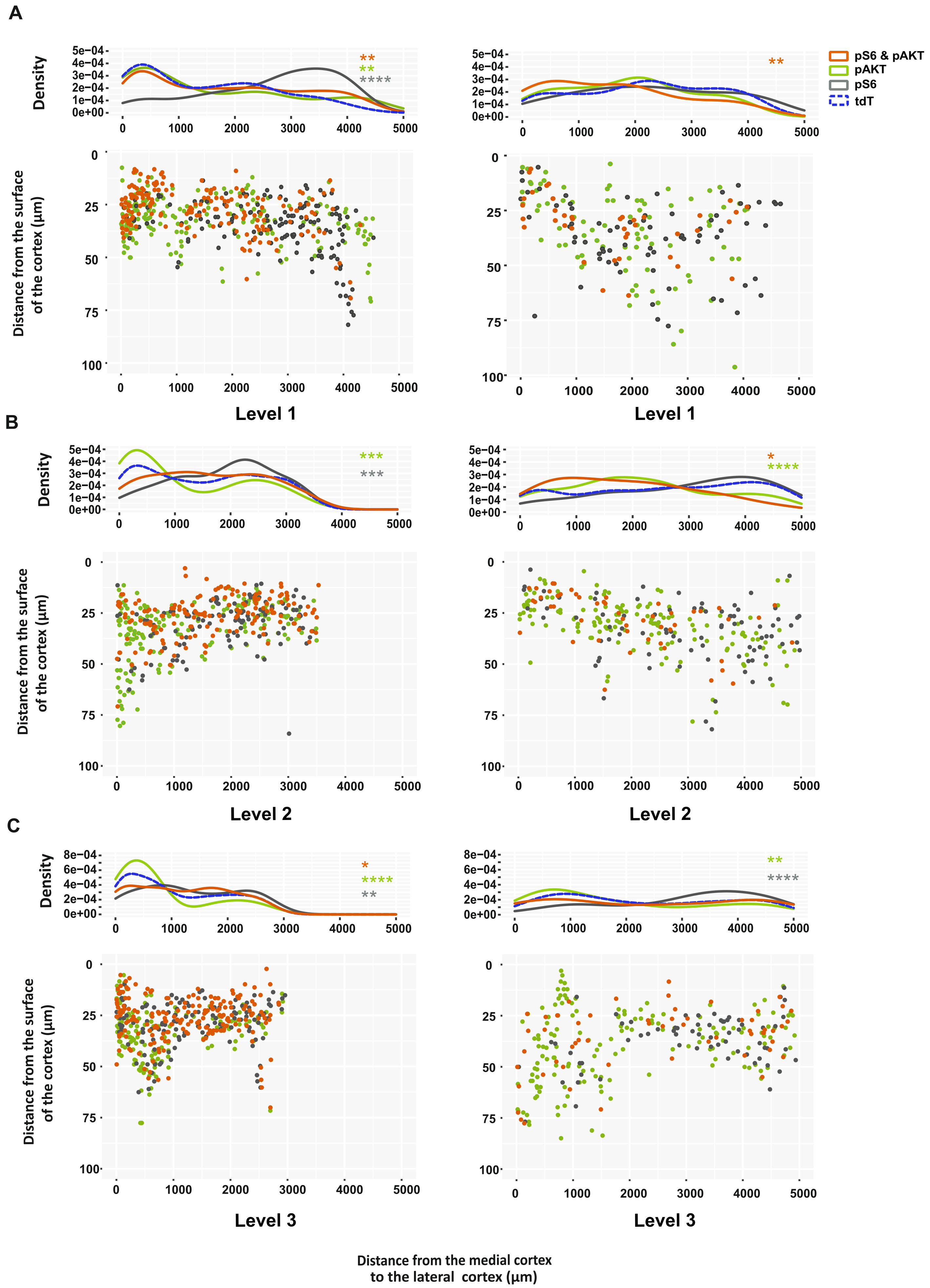

Figure S1 – Differential activity of the PI3K/AKT/mTOR pathway along the medio-lateral axis at 3 rostro-caudal levels in the neocortex at E17.5 and P1.
