# Supplementary material for: Activation of the PI3K/AKT/mTOR Pathway in Cajal–Retzius Cells Leads to Their Survival and Increases Susceptibility to Kainate-Induced Seizures
Source: Int J Mol Sci. 2023 Mar 11;24(6):5376. doi: 10.3390/ijms24065376 (PMC10048971; doi:10.3390/ijms24065376)
Supplement: Supplementary file 1 [file ijms-24-05376-s001.zip › Figure S2.pdf]

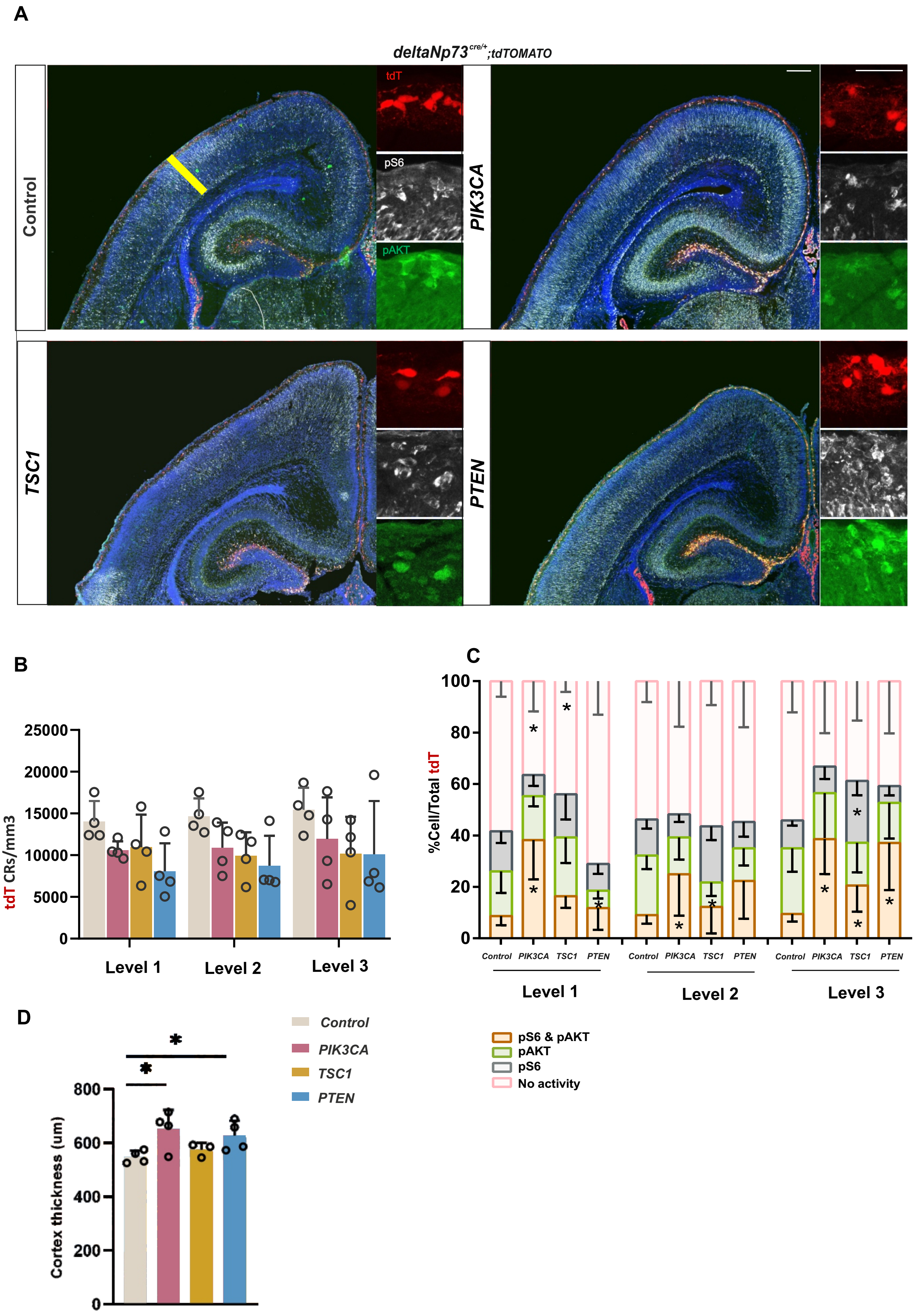

Figure S2- Differential activation of the PIK3/AKT/mTOR pathway at P1 does not alter CR development at P1 stage but cortical thickness in mutants with AKT activation.
