# Supplementary material for: Activation of the PI3K/AKT/mTOR Pathway in Cajal–Retzius Cells Leads to Their Survival and Increases Susceptibility to Kainate-Induced Seizures
Source: Int J Mol Sci. 2023 Mar 11;24(6):5376. doi: 10.3390/ijms24065376 (PMC10048971; doi:10.3390/ijms24065376)
Supplement: Supplementary file 1 [file ijms-24-05376-s001.zip › Figure S3.pdf]

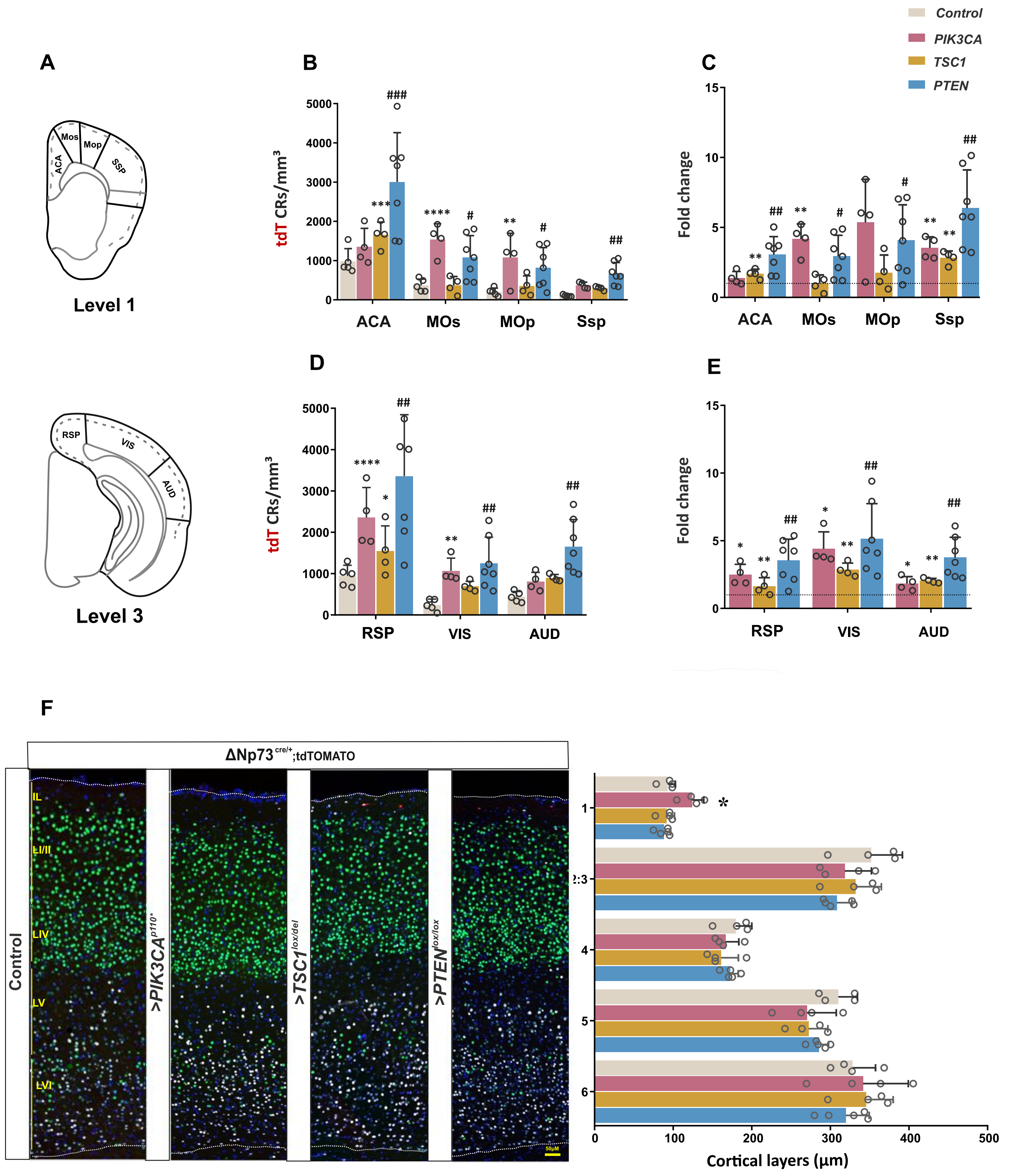

Figure S3 - Persitence of CRs at P24 along medio-lateral axe in levels 1 and 3 after PI3K/ AKT/ mTOR pathway activation . Normal cortical lamination in the S1bf brain area of the PIK3CA, TSC1 and PTEN mutants (Level 2) .
