# Supplementary material for: Activation of the PI3K/AKT/mTOR Pathway in Cajal–Retzius Cells Leads to Their Survival and Increases Susceptibility to Kainate-Induced Seizures
Source: Int J Mol Sci. 2023 Mar 11;24(6):5376. doi: 10.3390/ijms24065376 (PMC10048971; doi:10.3390/ijms24065376)
Supplement: Supplementary file 1 [file ijms-24-05376-s001.zip › Figure S4.pdf]

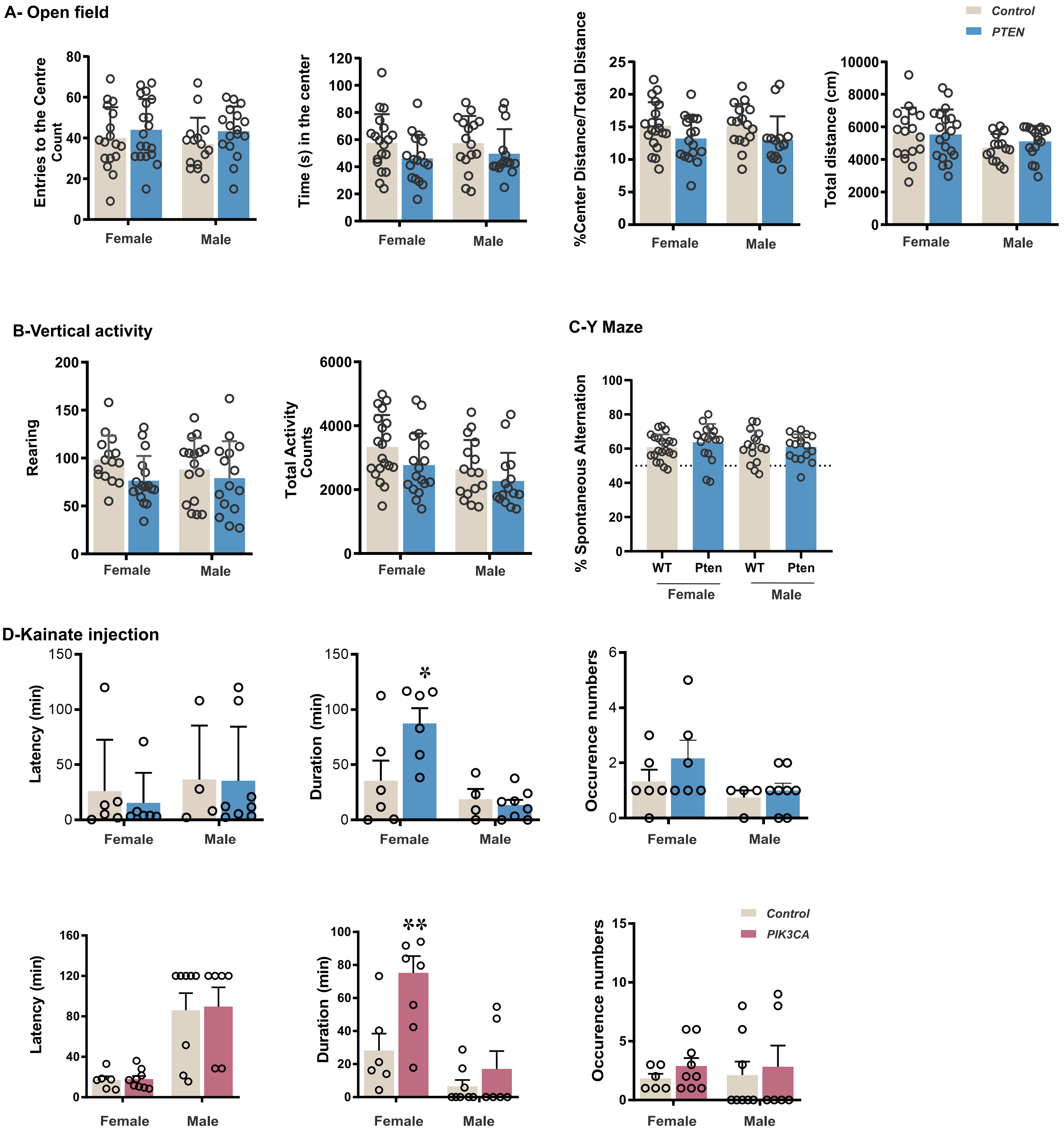

Figure S4_ Inactivation of PTEN in ΔNP73 derived CRs does not alter general animal behavior but increase the sensitivity of mutant females to kainate-induced seizures.
