# Supplementary material for: Activation of the PI3K/AKT/mTOR Pathway in Cajal–Retzius Cells Leads to Their Survival and Increases Susceptibility to Kainate-Induced Seizures
Source: Int J Mol Sci. 2023 Mar 11;24(6):5376. doi: 10.3390/ijms24065376 (PMC10048971; doi:10.3390/ijms24065376)
Supplement: Supplementary file 1 [file ijms-24-05376-s001.zip › Supplementary Figure Legends.pdf]

Supplementary Figure Legends :

Figure S1 – Differential activity of the PI3K/AKT/mTOR pathway along the medio-lateral axis at 3 rostro-caudal levels in the neocortex at E17.5 and P1. Kernel density (top) and scatter plots (bottom) of pS6 or pAKT positive CRs for levels 1 (A), 2 (B) and 3 (C) at E17.5 (left 6 panels) and P1 (right 6 panels) stages (n=4 for E17.5 and P1). Kolmogorov-Smirnov test (A, B, C). *p<0.05; **p<0.005, ***p<0.0005, ****p<0.0001.

Figure S2 – Differential activation of the PI3K/ AKT/ mTOR pathway at P1 does not alter CR development but cortical thickness in mutants with AKT activation. (A) Merged and single channel confocal images of P1 control brains (level 2) stained for pS6 Ser 240/244 (gray), pAKT Ser 473 (green) and Dapi (blue). tdTomato (red) traces ΔNp73 derived CRs. (B) CR density (CRs/mm3) at the neocortex levels 1, 2, and 3 (n=4 for controls and mutants). (C) Proportion of CRs positive for one or two markers or negative over total tdT cells (n=4 for controls and mutants) at the 3 levels. (D) Increased cortical thickness in future S1 area at P1 after AKT activation. Two-way ANOVA test followed by Sidak post-hoc test (B); Mann-Whitney U test (C); Unpaired t-test (D). Data are represented as mean ± SD; *p<0.05. Scale bars: 50 µm.

Figure S3 - Persistence of CRs at P24 along medio-lateral axes in levels 1 and 3 after PI3K/ AKT/ mTOR pathway activation. Normal cortical lamination in the S1bf brain area of the PIK3CA, TSC1 and PTEN mutants (Level 2). (A) Schematic representation of the two other rostro-caudal analyzed levels 1 and 3. (B, D) Quantification of CR densities (CRs/mm3) along medio-lateral axes of the neocortex levels 1 and 3 (C, E) Quantification of CR densities (fold changes) along medio-lateral axes of the neocortex levels 1 and 3. (F) Normal cortical lamination in the S1bf brain area (Level 2) of PI3KCA, TSC1 and PTEN mutants; Merged confocal images of con-trol and different mutants S1 barrel cortex at P24 stage stained for Cux1 (green), Ctip2 (white) markers and Dapi (blue), right panel: quantification of the layer thickness in different mutants and control. Two-way ANOVA followed by a Sidak's multiple comparisons test for Control vs PI3K and TSC1; Multiple t-test control vs PTEN (B, D) One sample t test (C,E) . Multiple t-test mutant vs. control (F); Data are represented as mean ± SD; *p<0.05; ** p<0.001; *** p<0.0001. # p<0.05; ## p<0.005; ### p<0.0005; #### p<0.0001; Scale bars: 50 µm

Figure S4 - Inactivation of PTEN in ΔNp73 derived CRs does not alter general animal behavior but increases susceptibility of mutant females to kainate-induced seizures. Anxiety in open-field (A), horizontal and vertical activities (A, B), spontaneous alternation in Y maze (C) were measured in control (grey) and Pten (blue) mutant males and females at 2 months and half stage. (D) Susceptibility to seizures after kainate injection was measured at 3 months by the la-tency, the duration and the number of seizures developed in the genetic context of Pik3/AKT/mTOR pathway activation by either the Pten inactivation (blue) or the Pik3CA (red) expression. Data are represented as mean ± SD; *p<0.05, ** p<0.008.
